# Contribution of Avian *Salmonella enterica* Isolates to Human Salmonellosis Cases in Constantine (Algeria)

**DOI:** 10.1155/2015/352029

**Published:** 2015-10-12

**Authors:** Rachid Elgroud, Sophie A. Granier, Muriel Marault, Annaëlle Kerouanton, Abdesslem Lezzar, Chafia Bouzitouna-Bentchouala, Anne Brisabois, Yves Millemann

**Affiliations:** ^1^Laboratory of Health Management and Animal Production, Veterinary Sciences Department, Constantine Mentouri University, Constantine, Algeria; ^2^Alfort National Veterinary School, Paris-Est University, 94704 Maisons-Alfort Cedex, France; ^3^Antimicrobial Resistance Unit, Maisons-Alfort Laboratory for Food Safety, ANSES, Paris-Est University, 94701 Maisons-Alfort Cedex, France; ^4^Hygiene and Quality of Poultry and Pig Products Unit, Anses Ploufragan Laboratory, European University of Brittany, 22440 Ploufragan, France; ^5^Service de Microbiologie-Bactériologie, C.H.U. Ibn Badis, Constantine, Algeria

## Abstract

An epidemiological investigation was carried out on one hundred *Salmonella* isolates from broiler farms, slaughterhouses, and human patients in the Constantine region of Algeria, in order to explore the contribution of avian strains to human salmonellosis cases in this region over the same period of time. The isolates were characterized by phenotypic as well as genotypic methods. A large variety of antimicrobial resistance profiles was found among human isolates, while only seven profiles were found among avian isolates. Enterobacterial Repetitive Intergenic Consensus-PCR (ERIC-PCR), Insertion Sequence *200*-PCR (IS*200*-PCR), and Pulsed Field Gel Electrophoresis (PFGE) resulted in the allocation of the isolates to 16, 20, and 34 different profiles, respectively. The 3 genotyping methods led to complementary results by underlining the clonality of some serovars with the diffusion and persistence of a single clone in the Constantine area as well as stressing the polymorphism present in isolates belonging to other serovars, indicating the diversity of potential reservoirs of nontyphoidal *Salmonella*. Altogether, our results seem to indicate that nontyphoidal avian *Salmonella* may play an important role in human salmonellosis in the Constantine region.

## 1. Introduction

*Salmonella* remains a major cause of illness in both humans and animals worldwide [1, 2]. It is estimated that* Salmonella* spp. are responsible for 93.8 million cases of human gastroenteritis and 155,000 deaths worldwide each year [3]. In the European Union, over 100,000 cases of salmonellosis were reported to EnterNet in 2003 [4] and over 90,000 cases in 2012, even though human salmonellosis cases have decreased regularly since 2005 [5]. It should be stressed that the observed reduction in salmonellosis cases is presumably the result of successful* Salmonella* control programmes in poultry populations [5].* Salmonella* is also a major public health concern in developing countries [6–8].

Salmonellosis due to nontyphoidal* Salmonella* is mainly associated with eating contaminated eggs, poultry meat, and pork. Contaminated poultry meat is identified as one of the principal sources of* Salmonella* in humans [2, 9]. Furthermore, one of the most frequent causes of infection by* Salmonella* reported in humans is the handling of raw poultry carcasses and products, together with the consumption of undercooked poultry meat [10].

The contamination of food products with* Salmonella* generates serious consequences for public health and the economy. This has motivated numerous studies designed to investigate the survival capacity of this bacterium and its transmission routes in farm-animals and their environment [11].

In the Constantine region (Algeria), a recent study showed that 37% of broiler farms and 53% of slaughterhouses were positive for* Salmonella* [12], with a predominance of* S*. Hadar,* S*. Virchow,* S*. Infantis,* S*. Albany, and *S*. Typhimurium. In a nearby region, 44% of laying hen flocks were reported to be positive for* Salmonella* [13].

In this study, we report on the epidemiological investigation of a certain number of serovars, isolated from broiler breeding farms, slaughterhouses, and human patients within the Constantine region.

Combined phenotypic and genotypic methods were used to assess the relationships between* Salmonella* strains isolated from these sources, in order to evaluate the contribution of avian strains to human salmonellosis in the region during the 2-year study. Phenotypic methods consisted of serotyping and antimicrobial susceptibility testing, whereas genotypic techniques were based on polymerase chain reaction (PCR) (i.e., Enterobacterial Repetitive Intergenic Consensus-PCR (ERIC-PCR) and Insertion Sequence* 200*-PCR (IS*200*-PCR)) and macrorestriction (i.e., Pulsed Field Gel Electrophoresis (PFGE)).

## 2. Materials and Methods

### 2.1. Bacterial Strains

For this study, we analysed 100 isolates recovered over a 2-year period (2006 through 2007) in the Constantine region (Table 1). The 45 human isolates studied (named H1 to H45) were obtained from the Constantine Hospital, whereas the 55 isolates of avian origin (named A1 to A55) were collected from poultry farms and slaughterhouses.

The isolation of avian strains was performed according to the NF U47-100 and NF U47-101 procedures [14, 15] at the Food Hygiene Laboratory from the Constantine Veterinary Sciences Department. Serotyping was carried out according to the White-Kauffmann-Le Minor scheme [16], as previously described [12].

### 2.2. Bacterial Susceptibility to Antibiotics

The antimicrobial susceptibility tests were performed using the disk diffusion method and interpreted as recommended by the “Comité de l'Antibiogramme de la Société Française de Microbiologie” [17]. Antimicrobials tested (load, breakpoints (mm)) were ampicillin (10 *μ*g, 19–14), amoxicillin-clavulanic acid (20/10 *μ*g, 21–14), cephalothin (30 *μ*g, 18–12), cefotaxime (30 *μ*g, 21–15), ceftazidime (30 *μ*g, 21–15), streptomycin (10 IU, 15–13), gentamicin (10 IU, 16–14), kanamycin (30 IU, 17–15), chloramphenicol (30 *μ*g, 23–19), tetracycline (30 IU, 19–17), sulphamethoxazole-trimethoprim (23.75 *μ*g + 1.25 *μ*g, 16–10), sulphonamides (200 *μ*g, 17–12), nalidixic acid (30 *μ*g, 20–15), ofloxacin (5 *μ*g, 22–16), enrofloxacin (5 *μ*g, 22–17), and colistin (50 *μ*g, 15). Zone diameters were read using the automated scanner Osiris (Bio-Rad).

### 2.3. PCR Methods

DNA was extracted by a boiling method as described previously [18]. The intergenic segments were amplified using the primers' sequences described by Millemann et al. [18] and Versalovic et al. [19]. All amplifications were performed on a Perkin Elmer 9700 thermal cycler (Courtaboeuf, France) as previously described [18].

### 2.4. PFGE Genotyping

PFGE was performed using a CHEF-DR III system (Bio-Rad, Marnes La-Coquette, France) according to the Salm-gene and PulseNet standardized protocol [20–22]. Two endonucleases were used,* Xba*I for all serovars and* Bln*I for* S*. Hadar. The* S. enterica* Braenderup H9812 strain was used as an internal control and molecular size marker [23]. DNA patterns were analysed with BioNumerics software (V 6.6, Applied Maths, Sint-Martens-Latem, Belgium). Dendrograms were produced using the Dice coefficient and an unweighted pair group method with arithmetic averages (UPGMA) with a 1% tolerance limit and 1% optimization (Pulsenet Europe recommendation [20]).

## 3. Results

*Salmonella* isolates were grouped into 16 different serovars (Table 1). Six serovars, namely, Agona, Anatum, Blockley, Indiana, Kentucky, and Senftenberg, were only recovered from humans during the two-year study, whereas 3 serovars, namely, Carnac, Montevideo, and Rissen, were only isolated from poultry. Isolates belonging to the 7 remaining serovars, that is, Albany, Enteritidis, Hadar, Heidelberg, Infantis, Typhimurium, and Virchow, were recovered from both poultry and humans.

**Figure 1:**
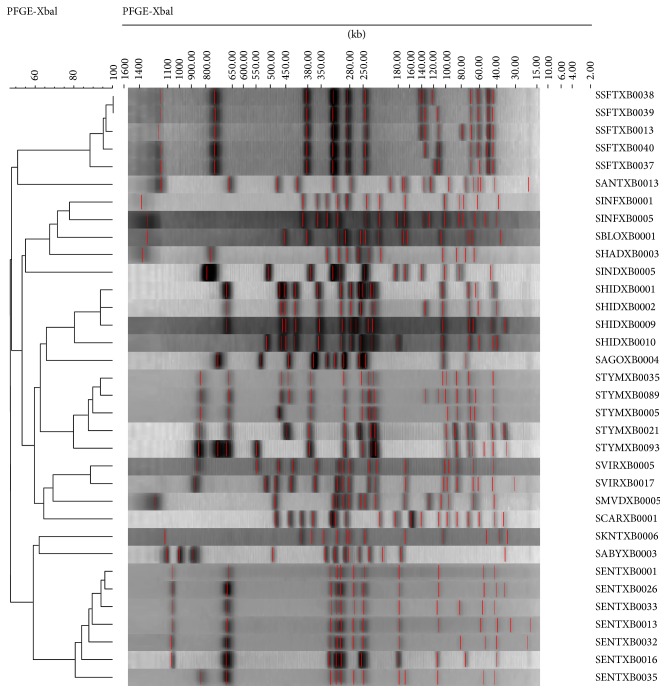
Dendrogram based on* Xba*I Pulsed Field Gel Electrophoresis (PFGE) profiles of major* Salmonella* serovars identified in Constantine (Algeria). Similarity percentages are figured on the left; names of the profiles are listed on the right. SABY:* Salmonella* Albany; SANT:* Salmonella* Anatum; SAGO:* Salmonella* Agona; SBLO:* Salmonella* Blockley; SCAR:* Salmonella* Carnac; SENT:* Salmonella* Enteritidis; SHAD:* Salmonella* Hadar; SHID:* Salmonella* Heidelberg; SIND:* Salmonella* Indiana; SINF:* Salmonella* Infantis; SKNT:* Salmonella* Kentucky; SMVD:* Salmonella* Montevideo; SSFT:* Salmonella* Senftenberg; STYM:* Salmonella* Typhimurium; SVIR:* Salmonella* Virchow.

A total of 16 ERIC-PCR, 20 IS*200*-PCR, 30 antimicrobial resistance, and 34 PFGE profiles were generated from the 100 isolates. For all isolates studied, those belonging to the same serovar clustered together (Table 1 and Figure 1).

The different ERIC-PCR profiles obtained were numbered from I to XVI and IS-PCR profiles were identified by the letters A through T (Table 1). Rissen and Infantis isolates shared the same ERIC- and IS*200*-PCR profiles.

The 34 different PFGE profiles obtained were numbered according to the preexisting database. Based on PFGE patterns, different situations were established among the analysed isolates, which led to various hypotheses. All PFGE results are shown in Table 1 and Figure 1.

## 4. Discussion

Evaluating the contribution of various animal sources to the burden of human salmonellosis is very difficult and requires microbial subtyping approaches [24] that depend on the comparison of the phenotypic and genotypic characteristics of the isolates studied. This consists of comparing serovars isolated from animals and humans to normal findings in both national and international serovar-based surveillance databases. Finally, the use of molecular markers for which there is a database may be also useful.

### 4.1. Serovars Isolated in Poultry and Humans

The serovars isolated from broilers in our study represent those usually present in broilers worldwide, especially in the USA and Europe [5, 25]. In our study, we recovered 6 serovars from broilers (i.e., on farms or in slaughterhouses) that are among the top 10 serovars encountered in Europe, including Enteritidis, Hadar, Indiana, Infantis, Typhimurium, and Virchow [5]. However, our study did not reflect this order as Hadar was isolated most frequently followed by Virchow, Infantis, and Albany.

Enteritidis and Typhimurium were the serovars most often isolated from human clinical cases in this study. This is generally consistent with other worldwide studies, for instance, in the USA and in Europe, as well as in Africa [5, 6, 25]. Senftenberg was ranked third, followed by Heidelberg, Blockley, and Kentucky. The high occurrence of Senftenberg is somewhat surprising and may be related to extensive commercial links with France. On the other hand one would have expected a slightly higher number of* S*. Kentucky isolates due to the recent emergence and distribution of this serovar in Africa [26, 27].

Interestingly, although it is rarely isolated from broiler and laying hen flocks, Albany was frequently isolated from broilers in our study [13, 26, 28]. Carnac is an extremely rare serovar in both poultry and humans. For instance, only one Carnac isolate was recovered from poultry in the European base line studies in 2008 [27] and the 2013 USA atlas for* Salmonella* did not report Carnac isolates for humans [29].

Some serovars (i.e., Agona, Anatum, Blockley, Indiana, Kentucky, and especially Senftenberg) were only isolated from humans in our study. However, those serovars are frequently isolated from various poultry species and are associated with chicken consumption when isolated in humans [28]. Senftenberg is mainly isolated in hatcheries and laying hen farms, and, in 2012, it ranked fourth among laboratory-confirmed* Salmonella* isolates from nonclinical nonhuman sources submitted to the National Veterinary Services Laboratories (NVSL) for typing in the USA [24]. This is one of the most commonly isolated serovars in France. For instance, in 2008,* S.* Senftenberg ranked first in total isolates collected from nonhuman sources as well as from poultry farm environments [28]. Kentucky is an emerging serovar in poultry and human and, recently, a particular multidrug resistant (MDR) phenotype has emerged in Africa and spread throughout poultry plants [30]. This MDR phenotype has also been isolated from laying hen flocks in Algeria [13]. Nevertheless, the Kentucky isolates from this study, although they were multidrug resistant, could not be linked to the global epidemic described by le Hello et al. [30] as these isolates are fully susceptible to fluoroquinolones.

Thus, considering the 7 serovars isolated in this study from both humans and poultry as well as the 6 serovars usually linked to human infection by poultry, isolates belonging to 13 of the 16 identified serovars suggest the potential link between poultry contamination and human salmonellosis.

### 4.2. Contribution of Epidemiological Markers to the Comparison of Avian Isolates and Human Isolates

Among the 7 serovars isolated from both humans and poultry in this study, 4 serovars (i.e., Albany, Hadar, Heidelberg, and Virchow) included human and avian isolates with indistinguishable patterns. In contrast, human and avian strain patterns did not match for serovars Enteritidis, Infantis, or Typhimurium.

#### 4.2.1. Matching Avian and Human Patterns

Serovar Albany strains were isolated from 3 different sources (i.e., humans, breeding farms, and slaughterhouses) but could not be differentiated by PFGE after digestion by restriction enzyme* Xba*I. There were only two strains of this serovar in the ANSES database and the identified profile SABYXB0003 was new. Therefore, it remains difficult to determine any genetic heterogeneity among these isolates. However, the two isolates from the slaughterhouses shared distinct ERIC-PCR and IS-PCR profiles. As a whole, our results suggest an epidemiological link between strains isolated from breeding farms, humans and, to a lesser extent, slaughterhouses. This conclusion is supported by the very similar antimicrobial resistance patterns observed, especially since fluoroquinolones were targeted.

Twenty-four Hadar isolates isolated from slaughterhouses, farms, and humans were characterized. All isolates merged with a single PFGE profile, with digestion by either* Xba*I or* Bln*I restriction enzymes, which seems to demonstrate the clonal character of the strains isolated from broiler chickens and humans. Nevertheless, we must be cautious since Hadar is considered to be a genetically homogeneous serovar (DI = 0.70 [20]). The comparison with the ANSES database showed that, with* Xba*I, 24 profiles had been identified out of the 153 strains of this previously studied serovar and the DI was only 0.48. This possible epidemiological link also seems to be supported by the single profile found by ERIC-PCR and the IS-PCR profile, with the exception of 2 strains isolated from slaughterhouses. The 2 dissimilar Hadar isolates were associated with turkeys slaughtered in the same slaughterhouse. Antibiotyping also gave a different reading in that human isolates were multiresistant and therefore differentiated, whereas all the other isolates shared a single resistance pattern.

For Heidelberg and Virchow, we identified at least one common pulsotype in avian and human isolates, which may indicate an avian source for human infection. Additionally, the SHIDXB0001 profile, identified in a human Heidelberg strain, had previously been found in the poultry chain.

Two different PFGE profiles were identified for the Virchow isolates. It is possible that isolates exhibiting a SVIRXB0005 profile may have spread from broiler chickens to consumers. This hypothesis is supported by our results where all strains isolated from slaughterhouses shared this profile. To date, 93 strains of this serovar have been recorded in the database and 24 different profiles have been identified.

#### 4.2.2. Nonmatching Human and Avian Patterns

Although human illnesses due to Enteritidis, Infantis, Senftenberg, and Typhimurium are commonly linked to avian sources, we did not find any matching pulsotypes between the avian and human isolates of these serovars. This must be emphasized particularly for Enteritidis and Senftenberg, even though they tend to originate in laying hens rather than broilers [28, 31, 32]. However, Cardinale et al. [33] highlighted the genetic similarity of* S*. Enteritidis PFGE profiles from human and broiler sources in Senegal. We may add that the SENTXB0001 profile has already been encountered in isolates of human origin, as well as from poultry, pastries, cooked meals, sea products, and so forth.

## 5. Conclusion 

Our study did not confirm an association between the main serotypes detected in humans and those recovered in poultry production. However, collectively, our results bring to light a probable significant contribution of nontyphoidal* Salmonella* by avian species to human salmonellosis in the Constantine region. Since the majority of isolates belonged to serovars usually associated with poultry, and despite the very low number of isolates studied, we were able to confirm identical profiles among avian and human isolates. The development of a large monitoring programme is crucial for the surveillance of* Salmonella* in poultry and the improvement of public health in Algeria.

## Figures and Tables

**Table 1:** Characteristics of the avian and human studied isolates.

Strains	Serovars	ERIC-PCR	IS-PCR	Antimicrobial resistance pattern^*^	PFGE profile	Sources
H17	*S*. Agona	I	A	AM, CEF, CTX, SXT, SSS, GEN, STR	SAGOXB0004	Human

H45	*S*. Albany	II	B	NAL, OFX, STR	SABYXB0003	Human
A81, A88, A89				NAL, OFX, STR	SABYXB0003	Farm
A80				ENR, NAL, OFX, STR	SABYXB0003	Farm
A85, A86		III	C	NAL, OFX, STR	SABYXB0003	Slaughter

H38	*S*. Anatum	IV	D	Susceptible	SANAXB0013	Human

H21	*S*. Blockley	V	E	NAL	SBLOXB0001	Human
H41				Susceptible	SBLOXB0001	Human

A78, A79, A90, A91	*S*. Carnac	VI	F	Susceptible	SCARXB0001	Farm

H9	*S*. Enteritidis	VII	G	AMP, CAZ, SSS, TET	SENTXB0026	Human
H11				Susceptible	SENTXB0026	Human
H3, H14, H31, H32, H47				NAL, OFX	SENTXB0001	Human
H10				AMP, CTX, SSS, TET	SENTXB0001	Human
H25				NAL	SENTXB0001	Human
H26				NAL	SENTXB0013	Human
A82, A87				Susceptible	SENTXB0016	Human
H1				AMP, NAL	SENTXB0035	Human
H24				NAL, OFX	SENTXB0032	Slaughter
H2				NAL	SENTXB0033	Human
H7			H	NAL, OFX	SENTXB0001	Human

H4	*S*. Hadar	VIII	I	ENR, NAL, OFX, STR, TET	SHADXB0003	Human
H5				KAN, NAL, OFX, STR, TET	SHADXB0003	Human
H18, H44				AMP, NAL, OFX, STR, TET	SHADXB0003	Human
A28, A29, A30, A31, A32, A56				STR, TET	SHADXB0003	Slaughter
A36, A37, A38, A39				STR, TET	SHADXB0003	Farm
A33, A34, A35, A40,				STR, TET	SHADXB0003	Farm
A41, A42, A43, A44				STR, TET	SHADXB0003	Farm
A26, A27			J	STR, TET	SHADXB0003	Slaughter

H13	*S*. Heidelberg	IX	K	NAL, OFX	SHIDXB0002	Human
A60				NAL, OFX, STR	SHIDXB0002	Farm
H17				NAL	SHIDXB0009	Human
H23				NAL, OFX	SHIDXB0010	Human
H33				NAL, OFX	SHIDXB0001	Human

H46	*S*. Indiana	X	L	Susceptible	SINDXB0005	Human

A22, A23, A24, A25	*S*. Infantis	XI	M	NAL	SINFXB0001	Farm
A48, A49				Susceptible	SINFXB0001	Farm
H12				Susceptible	SINFXB0005	Human

H27, H28	*S*. Kentucky	XII	N	AMP, CAZ, CEF, CTX, GEN, KAN, SSS, SXT	SKNTXB0006	Human

A67	*S*. Montevideo	XIII	O	Susceptible	SMVDXB005	Slaughter

A21	*S*. Rissen	XI	P	NAL	—	Farm

H34	*S*. Senftenberg	XIV	Q	NAL, STR	SSFTXB0039	Human
H15				AM, CAZ, CF, CTX, GM, K, NAL, S, SSS	SSFTXB0013	Human
H30				NAL, STR	SSFTXB0038	Human
H35, H16			R	NAL, STR	SSFTXB0037	Human
H37				NAL, STR	SSFTXB0040	Human

H29	*S*. Typhimurium	XV	S	NAL	STYMXB0093	Human
H19, H20, H22, H36				AMP, CHL, SSS, STR, TET	STYMXB0035	Human
H8				NAL	STYMXB0089	Human
H6				NAL	STYMXB0005	Human
A17, A18, A45, A46			T	STR	STYMXB0021	Slaughter
A19				NAL, STR	STYMXB0021	Slaughter

A63	*S*. Virchow	XVI	U	Susceptible	SVIRXB0017	Farm
A66				NAL	SVIRXB0017	Farm
H40				Susceptible	SVIRXB0005	Human
A20				NAL	SVIRXB0005	Slaughter
A53, A65				Susceptible	SVIRXB0005	Slaughter
A62, A64, A77, A92				Susceptible	SVIRXB0005	Farm

^*^Susceptible: susceptible to all tested antibiotics. AMP: ampicillin; AMC: amoxicillin-clavulanic acid; CAZ: ceftazidime; CEF: cephalothin; CHL: chloramphenicol; CST: colistin; CTX: cefotaxime; ENR: enrofloxacin; GEN: gentamicin; K: kanamycin; NAL: nalidixic acid; OFX: ofloxacin; SSS: sulphonamides; STR: streptomycin; SXT: trimethoprim-sulfamethoxazole; TET: tetracycline.
